# The Role of ARF Family Proteins and Their Regulators and Effectors in Cancer Progression: A Therapeutic Perspective

**DOI:** 10.3389/fcell.2020.00217

**Published:** 2020-04-21

**Authors:** Cristina Casalou, Andreia Ferreira, Duarte C. Barral

**Affiliations:** CEDOC, NOVA Medical School, Faculdade de Ciências Médicas, Universidade NOVA de Lisboa, Lisbon, Portugal

**Keywords:** ARL, migration, invasion, tumorigenesis, guanine nucleotide exchange factor, GTPase-activating protein, membrane traffic

## Abstract

The Adenosine diphosphate-Ribosylation Factor (ARF) family belongs to the RAS superfamily of small GTPases and is involved in a wide variety of physiological processes, such as cell proliferation, motility and differentiation by regulating membrane traffic and associating with the cytoskeleton. Like other members of the RAS superfamily, ARF family proteins are activated by Guanine nucleotide Exchange Factors (GEFs) and inactivated by GTPase-Activating Proteins (GAPs). When active, they bind effectors, which mediate downstream functions. Several studies have reported that cancer cells are able to subvert membrane traffic regulators to enhance migration and invasion. Indeed, members of the ARF family, including ARF-Like (ARL) proteins have been implicated in tumorigenesis and progression of several types of cancer. Here, we review the role of ARF family members, their GEFs/GAPs and effectors in tumorigenesis and cancer progression, highlighting the ones that can have a pro-oncogenic behavior or function as tumor suppressors. Moreover, we propose possible mechanisms and approaches to target these proteins, toward the development of novel therapeutic strategies to impair tumor progression.

## Introduction

The Adenosine diphosphate-Ribosylation Factor (ARF) family of proteins belongs to the RAS superfamily of small GTPases and comprises around 30 members in mammals (Sztul et al., 2019). This family includes 6 ARFs (5 in humans since ARF2 is absent), 21 ARLs, 2 Secretion-Associated RAS-related (SARs) and the TRIpartite Motif-containing protein 23 (TRIM23). ARF1-5 regulate vesicle budding at the Golgi apparatus by recruiting coat complexes (Li et al., 2004; Kahn et al., 2006). ARF6 localizes to the plasma membrane, as well as endosomes and is involved in actin cytoskeleton dynamics and endocytic recycling (Donaldson, 2003). The functions of ARL proteins are more heterogeneous and currently unknown for several of them. ARL2 and ARL3 interact with microtubules and function in tubulin assembly and cytokinesis, respectively, while ARL4C and ARL4D are involved in actin remodeling and regulate cell migration (Li C.-C. et al., 2007; Chiang et al., 2017). Our laboratory has shown that ARL13B binds actin and regulates cell migration (Barral et al., 2012; Casalou et al., 2014). Interestingly, several ARLs, namely ARL3, ARL6 and ARL13B are associated with the cilium and play different roles in ciliary biology and signaling pathways associated with this organelle (Marwaha et al., 2019). ARL8B is well characterized and has been shown to localize to lysosomes and regulate several aspects of lysosome biology, such as positioning and motility (Khatter et al., 2015). Finally, SARs play a well-described role in the budding of COPII-coated vesicles from the ER, while TRIM23 was implicated in antiviral defense and adipocyte differentiation (Arimoto et al., 2010; Watanabe et al., 2015; Saito et al., 2017).

Like other GTPases, ARF family proteins switch between an active state, in which proteins are GTP-bound and an inactive state, in which proteins are GDP-bound. For this reason, they are referred to as “molecular switches.” Nucleotide exchange is catalyzed by GEFs and GTP hydrolysis is promoted by GAPs. When they are active, ARF proteins associate with membranes via lipid modifications, namely myristoylation, palmitoylation or acetylation and bind effectors. These are responsible for the downstream functions of ARF family proteins and are highly diverse. Among the effectors identified are coat complexes and adaptors, cytoskeleton-binding proteins and tethering factors (Donaldson and Jackson, 2012). The functions of ARF and ARL proteins, as well as their GEFs and GAPs are thoroughly reviewed in two excellent recent reviews (Marwaha et al., 2019; Sztul et al., 2019).

Since ARFs and their regulators play essential functions in cell cycle, cytoskeleton remodeling, cell migration and adhesion, it is not surprising that they can be subverted by cancer cells for proliferation, migration and invasion. Indeed, the expression and/or activity of several ARF family proteins and their GEFs and GAPs has been shown to be modulated in several types of cancer (Tables 1, 2). Moreover, the amplification and overexpression of ARF family genes, as well as the overexpression of their GEFs and GAPs, and variance in post-translational modifications are the most commonly detected alterations thought to be implicated in cancer. Here, we review the members of the ARF family and their activity regulators and effectors that have been implicated in cancer, and can either function as oncogenes or tumor suppressors and propose possible therapeutic approaches to target ARF family proteins or their effectors, GEFs and GAPs.

**TABLE 1:** Expression of ARF family members in human neoplastic tissues and cancer cells.

**ARF/ARL**	**Expression**	**Cancer type**	**References**
ARF1	**↑**	Breast, Colon/Colorectal, Gastric, Liver, Ovarian, Osteosarcoma, Prostate	Olstad et al., 2003; Ma et al., 2005; Kannangai et al., 2007; Tsai et al., 2012; Schlienger et al., 2015; Davis et al., 2016; Gu et al., 2017
ARF3	**↑**	Breast	Huang et al., 2019
	**↓**	Gastric	Chang et al., 2009
ARF4	**↑**	Breast, Glioma, Lung, Ovarian	Woo et al., 2009; Bidkhori et al., 2013; Howley et al., 2018; Wu Q. et al., 2018
ARF6	**↑**	Breast, Gastric, Glioma, Liver, Lung, Melanoma, Pancreatic, Prostate, Renal Cell Carcinoma	Hashimoto et al., 2004; Hu et al., 2009; Knizhnik et al., 2011; Oka et al., 2014; Morgan et al., 2015; Zhang et al., 2015; Hashimoto et al., 2016; Liang et al., 2017; Qi et al., 2019; Yoo et al., 2019
ARL2	**↑**	Bladder, Cervical, Liver	Hass et al., 2016; Li H.-J. et al., 2017; Peng et al., 2017
	**↓**	Breast	Beghin et al., 2009
ARL3	**↓**	Glioma	Wang et al., 2019b
ARL4C	**↑**	Colon/Colorectal, Gastric, Glioma, Head and Neck, Liver, Lung, Muscle, Renal Cell Carcinoma	Fujii et al., 2014; Guo et al., 2015; Fujii et al., 2016; Hu et al., 2018; Chen et al., 2019; Harada et al., 2019; Isono et al., 2019
	**↓**	Ovarian	Su et al., 2015
ARL4D	**↑**	Glioma	Chi et al., 2008
ARL5A	**↑**	Colon/Colorectal	Wang et al., 2014
ARL6	**↑**	Muscle	Liu et al., 2016
ARL11	**↓**	Breast, Leukemia, Lung, Ovarian, Prostate	Calin et al., 2005; Petrocca et al., 2006; Yendamuri et al., 2008; Siltanen et al., 2013
ARL13B	**↑**	Breast, Gastric	Shao et al., 2018; Casalou et al., 2019
ARL14	**↑**	Bladder	Wang L. et al., 2019
ARFRP1	**↑**	Gastric	Mao et al., 2018
TRIM23	**↑**	Gastric	Yao et al., 2018
SARI A	**↑**	Liver	He et al., 2002
SAR1B	**↓**	Colon/Colorectal	Huang and Wang, 2019

*(↑), upregulated; (↓), downregulated.*

**TABLE 2:** Expression of ARF GEFs in human neoplastic tissues and cancer cells.

**ARF GEF/GAP**	**Expression**	**Cancer type**	**References**
Cytohesin 1	**↑**	Leukemia/Lymphoma	Villalva et al., 2002
Cytohesin 2	**↑**	Colon/Colorectal, Liver	Xu et al., 2013; Pan et al., 2014; Qi et al., 2019
Cytohesin 3	**↑**	Liver	Fu et al., 2014
BRAG2	**↑**	Breast, Lung, Pancreatic	Hiroi et al., 2006; Morishige et al., 2008; Menju et al., 2011; Oka et al., 2014
BIG2	**↑**	Pancreatic	Park et al., 2011
EFA6	**↑**	Glioma, Renal Cell Carcinoma	Li et al., 2006; Hashimoto et al., 2016
	**↓**	Breast, Brain, Ovarian	Pils et al., 2005; Van Den Boom et al., 2006; Zangari et al., 2014
ASAP1	**↑**	Bladder, Breast, Colon/Colorectal, Esophagus, Gastric, Head and Neck, Melanoma, Ovarian, Pacreatic, Prostate, Renal Cell Carcinoma, Thyroid	Ehlers et al., 2005; Onodera et al., 2005; Lin et al., 2008; Müller et al., 2010; Hou et al., 2014; Li et al., 2014; Sato et al., 2014
	**↓**	Cervical	Müller et al., 2010
ASAP3	**↑**	Liver, Lung, Ovarian	Okabe et al., 2004; Fan et al., 2014; Willis et al., 2016
AGAP1	**↑**	Leukemia/Lymphoma	Harvey et al., 2010
AGAP2	**↑**	Bladder, Breast, Brain, Cervical, Colon/Colorectal, Gastric, Glioma, Head and Neck, Leukemia/Lymphoma, Liver, Lung, Ovarian, Prostate	Ahn et al., 2004; Knobbe et al., 2005; Van Den Boom et al., 2006; Liu et al., 2007; Cai et al., 2009; Xie et al., 2012; Doush et al., 2019
GIT1	**↑**	Breast, Cervical, Colon/Colorectal, Head and neck, Liver, Lung, Melanoma, Renal Cell Carcinoma	Yoo et al., 2012; Chan et al., 2014; Huang et al., 2014; Peng et al., 2014; Chang et al., 2015; Lu et al., 2016
GIT2	**↓**	Breast	Sirirattanakul et al., 2015
SMAP1	**↓**	Colon/Colorectal	Sangar et al., 2014
ARFGAP3	**↑**	Prostate	Nalla et al., 2016
ARAP3	**↓**	Gastric	Yagi et al., 2011

*(↑), upregulated; (↓), downregulated.*

## ARF Family Proteins and Their Activity Regulators and Effectors That Can Function as Oncogenes

Dysregulation of expression and/or activity of ARF family proteins and/or their effectors, GEFs and GAPs has been associated with enhanced cell migration, invasion and proliferation in several types of cancer. In this section, we review the ARF family members, as well as their activity regulators and effectors that have been found overexpressed in cancer and play essential roles in cancer progression (Tables 1, 2).

### ARF1

ARF1 plays a central role in maintaining the structure and function of the Golgi apparatus and is highly expressed in breast, prostate and ovarian cancers (Schlienger et al., 2015; Davis et al., 2016; Gu et al., 2017). In the context of cancer, ARF1 has an important function in inter- and intracellular signaling, cell cycle regulation and DNA repair, as well as necrosis and apoptosis (D’Souza-Schorey and Chavrier, 2006; Gu et al., 2017). Moreover, ARF1 regulates breast cancer cell adhesion and proliferation, being essential for EGF-mediated phosphorylation of Focal Adhesion Kinase (FAK) and Src (Schlienger et al., 2015). Furthermore, ARF1 sensitizes MDA-MB-231 breast cancer cells to the anti-tumor drugs actinomycin D and vinblastine through ERK and Akt signaling (Luchsinger et al., 2018). In prostate cancer, ARF1 promotes tumorigenesis by controlling MAPK activation and cell growth (Davis et al., 2016). In myeloma cells, ARF1 expression promotes cell proliferation and inhibits cell adhesion, controlling proliferation- and cell adhesion-mediated drug resistance (Xu et al., 2017). Finally, ARF1 is upregulated in ovarian tumors, when compared with adjacent non-cancerous tissues and its overexpression is associated with ovarian cancer cell proliferation and migration through the PhosphoInositide 3-Kinase (PI3K) pathway (Gu et al., 2017).

### ARF3

Like ARF1, ARF3 is involved in the recruitment of coat complexes to the Golgi apparatus, activation of PhosphoLipase D (PLD) and PI-kinases. Recently, ARF3 expression was positively correlated with breast cancer clinical stages, being upregulated in 92.8% of malignant cases, relative to benign ones (Huang et al., 2019). Indeed, ARF3 mRNA and protein expression levels are upregulated in human breast cancer cell lines and tissues (Huang et al., 2019). Moreover, ARF3 overexpression promotes breast cancer cell proliferation by regulating the cell-cycle G1/S transition, through inhibition of FOXO1 transcription factor activity (Huang et al., 2019). Additionally, *ARF3* was found to be a candidate gene involved in the progression of pregnancy-associated breast cancer, based on integrated analysis of microarray profile datasets (Zhang et al., 2019).

### ARF4

Together with the upregulation of *COPB1* and *USO1*, which encode for the COPI subunit β1 and General vesicular transport factor p115, respectively and regulate ER-Golgi trafficking, ARF4 has been reported to be upregulated in breast cancer patient samples (Howley et al., 2018). This establishes a role for *ARF4*, *COPB1*, and *USO1* in the regulation of breast cancer cell growth and invasion through the retrograde transport of proteins from the Golgi to ER via COPI-coated vesicles. ARF4 has also been associated with the regulation of breast cancer cell migration in response to Phorbol-12-Myristate 13-Acetate (PMA) (Jang et al., 2012). Finally, ARF4 has been found upregulated in other types of epithelial cancers, such as ovarian cancer (Wu Q. et al., 2018) and lung adenocarcinomas (Bidkhori et al., 2013). In U373MG human glioblastoma-derived cells, ARF4 has an anti-apoptotic function by reducing the generation of ROS in response to the expression of B-cell lymphoma 2 (Bcl-2)-Associated X protein (Bax) or the synthetic retinoid derivative N-(4-hydroxyphenyl) retinamide (Woo et al., 2009).

### ARF6

ARF6 is well characterized in the context of cancer and known to regulate cancer cell invasion and metastasis, as well as tumor angiogenesis and growth (reviewed in Hongu et al., 2016; Li R. et al., 2017). Clinically, ARF6 expression and activation of its downstream signaling pathways was determined and associated with poor overall survival of breast, lung adenocarcinoma, pancreatic ductal adenocarcinoma and head and neck cancer patients (Li R. et al., 2017). Also, elevated ARF6 expression has been reported in prostate and non-small cell lung and squamous cell lung cancers (Knizhnik et al., 2011; Morgan et al., 2015). Moreover, a direct correlation between ARF6 protein expression levels and breast cancer cell invasiveness was shown in breast cancer cell lines with different invasive abilities (Hashimoto et al., 2004). Furthermore, ARF6 silencing impairs invasion of breast cancer, melanoma and glioma (Hashimoto et al., 2004; Hu et al., 2009; Grossmann et al., 2014), providing evidence that ARF6 is an important driver of cancer cell invasion and metastasis. In lung adenocarcinoma, the combined expression of ARF6, its GEF BRAG2/GEP100 and EGFR is associated with decreased patient survival (Oka et al., 2014). ARF6 is known to recruit actin binding proteins, adhesion molecules and proteases, which are essential for invadopodia formation and ExtraCellular Matrix (ECM) degradation (Schweitzer et al., 2011). Indeed, ARF6 activation was shown to promote invadopodia formation through activation of Rho- and Rac1-dependent pathways (Muralidharan-chari et al., 2009). ARF6 is also necessary for Human Growth Factor (HGF)-induced tumor angiogenesis and growth (Hongu et al., 2015). It has also been shown that ARF6 coordinates signaling and function of several oncogenes, like *EGFR*, *ERBB2*, and *CTNNB1*, which encode for EGFR, HER2, and β-catenin, respectively (Morishige et al., 2008; Menju et al., 2011; Pellon-Cardenas et al., 2013; Yoo et al., 2016). In agreement, it was recently observed that ARF6 is a downstream target of mutant KRAS and maintains KRAS-induced ERK activation, promoting pancreatic tumorigenesis (Liang et al., 2017). Also, ARF6 was linked to liver cancer through the regulation of the endocytic recycling of CD147, a tumor-related adhesive protein that promotes invasion of liver cancer cells (Qi et al., 2019). Moreover, the increased expression of ARF6-CD147 signaling components, like Cytohesin 2/ARNO, an ARF6 GEF and Rac1 were associated with poor overall survival of hepatocellular carcinoma patients (Qi et al., 2019).

### ARF GEFs and GAPs

Amplification of ARF GAPs has been associated with several types of cancer. Indeed, AGAP2, which acts on ARF1 and ARF5, promotes cancer cell survival, migration and invasion in gliobastomas (Qi et al., 2017). Moreover, ASAP1 expression is correlated with the metastatic potential of melanoma, prostate cancer and colorectal cancer and increased invasiveness of breast cancer and melanoma cells (Ehlers et al., 2005; Onodera et al., 2005; Lin et al., 2008; Müller et al., 2010). Cancer cell migration requires coordinated assembly and disassembly of cell-ECM contacts, mediated by FAs. Indeed, several ARF GAPs, namely ASAP1, ASAP2, GIT1 and GIT2 have been found to be localized at FAs (Casalou et al., 2016). GIT1, which inactivates ARF6 specifically, is highly expressed in several types of cancers, including breast, cervical, colon and liver (Yoo et al., 2012; Chan et al., 2014; Huang et al., 2014; Peng et al., 2014). Moreover, GIT1 interacts with Paxillin and p21-activated kinase Interacting eXchange factor (PIX) at FAs, regulating cancer cell migration (Nayal et al., 2006). Furthermore, GIT1 silencing has been shown to inhibit cell migration and invasion in oral squamous cell carcinoma and breast cancer (Chan et al., 2014; Huang et al., 2014). Although GIT1 is associated with several types of cancer, it is not clear whether ARF6 inactivation by GIT1 is a requirement for cancer progression.

Regarding the ARF GEFs, BRAG2/GEP100 and EFA6, which activate ARF6 specifically, are known to be involved in cancer progression. BRAG2 induces breast cancer cell invasion and metastasis (Morishige et al., 2008). After BRAG2 binding to phosphorylated EGFR, ARF6 is activated in breast cancer cells, leading to the formation of invadopodia with recruitment of Cortactin, Paxillin and the ARF GAP ASAP1 (Onodera et al., 2005; Morishige et al., 2008). In lung adenocarcinoma, the pathway involving EGFR, ARF6 and ASAP1 was reported to be associated with reduced patient survival (Oka et al., 2014). In melanoma cells, the stimulation of WNT5A, a member of the Wnt signaling pathway, induces ARF6 activation mediated by BRAG2, which facilitates the release of β-catenin from cadherin and stimulates tumor cell invasion (Grossmann et al., 2014).

Concerning the EFA6 GEFs, they regulate tumor progression either positively or negatively, depending on the cancer types. In glioma and renal carcinoma, EFA6 GEFs are upregulated, controlling cancer cell invasion (Li et al., 2006; Hashimoto et al., 2016). The ARF GEFs Cytohesin 1-3 function as regulators of cytoskeleton reorganization and integrin signaling (Kolanus, 2007) and target ARF6, among other ARFs. In prostate cancer, inhibiton of Cytohesin 1 by siRNA, reduces the pro-tumorigenic role of Insulin Growth Factor Receptor (IGFR) signaling (Weizhong et al., 2011), suggesting that this ARF GEF could be targeted to impair prostate cancer progression. Additionally, the ectopic expression of the constitutively active form of ARF6 (ARF Q67L) enhances melanoma progression and metastasis *in vivo* (Muralidharan-chari et al., 2009).

### ARL2

ARL2 was first reported to behave as a tumor suppressor in breast cancer. However, several publications thereafter suggest that this might not be the case for other types of cancers. Indeed, it was shown that BART binds to active ARL2, inhibiting the inactivation of RhoA and thus impairing the invasive potential of pancreatic cancer cells (Taniuchi et al., 2011). Other studies evaluated the effect of ARL2-targeting microRNAs (miRs). In particular, miR-214 was found to suppress growth and increased apoptosis in colon cancer (Long et al., 2015). Moreover, miR-214 was studied in the context of cervical cancer, in which its expression is able to suppress proliferation, migration and invasion of cancer cells (Peng et al., 2017). Two other miRs were found to be involved in cancer progression. miR-497-5p overexpression leads to a decrease in osteosarcoma cell proliferation and an increase in apoptosis (Sun et al., 2017). On the other hand, miR-195, which is regulated by Urothelial Cancer Associated 1 (UCA1) targets ARL2 in bladder cancer (Li H.-J. et al., 2017). Studies performed in mice showed that bladder tumor size is reduced upon UCA1 downregulation and the expression of miR-195 is increased, resulting in ARL2 downregulation. The authors concluded that the effects in bladder cancer cells mediated by UCA1/miR-195/ARL2 are a consequence of mitochondrial metabolism modulation, which regulates cancer cell survival (Li H.-J. et al., 2017). Finally, *ARL2* was found to be overexpressed in human hepatocellular carcinoma samples by gene expression analysis (Hass et al., 2016).

### ARL4

*ARL4C* was initially found to be upregulated at the mRNA level in both colorectal and lung cancers (Fujii et al., 2014). Moreover, the same authors found that ARL4C silencing leads to a decrease in cell migration and invasion *in vitro*, and proliferation both *in vitro* and *in vivo*, dependently on aberrant Wnt/β-catenin and EGF/RAS signaling. ARL4C was also found to be overexpressed in leiomyosarcoma type II (Guo et al., 2015). Furthermore, analysis of ARL4C expression in colorectal cancer samples revealed that this ARL is more expressed in tumor samples, comparing with adjacent normal tissue (Chen et al., 2016). The prognostic value of ARL4C in colorectal cancer was also evaluated and the same authors concluded that patients with higher expression of ARL4C have lower survival on average (Chen et al., 2016). In the case of lung and tongue squamous cell carcinoma, it was found that ARL4C promotes proliferation and migration of cells from these types of cancers (Fujii et al., 2016). Interestingly, ARL4C overexpression in lung tumors was shown to be due to hypomethylation of *ARL4C* in the 3′-UTR through Ten-Eleven Translocation methylcytosine dioxygenases (TETs) (Fujii et al., 2016). Recently, several groups investigated the role of ARL4C in different types of cancer. *ARL4C* was identified as a peritoneal dissemination-associated gene and found to be highly expressed in gastric cancer cells (Hu et al., 2018). Indeed, ARL4C silencing impairs migration and invasion of gastric cancer cells *in vitro* (Hu et al., 2018). Moreover, the reduced expression of ARL4C leads to a decrease of the epithelial-mesenchymal transition (EMT) marker SLUG, as well as a reduction in lamellipodia and filopodia formation in gastric cancer cells (Hu et al., 2018). ARL4C expression was also found to be increased in primary and metastatic hepatocellular carcinoma. Additionally, the decrease in ARL4C expression leads to the impairment of cancer cell proliferation and migration *in vitro* and *in vivo*, as well as a reduction in expression of PI3K Catalytic subunit Delta (*PI3KCD*) mRNA and activity of Akt (Harada et al., 2019). This suggests that the molecular mechanisms involved in the role of ARL4C in hepatocellular carcinoma are different from those in lung and colorectal cancers. Furthermore, upregulation of ARL4C was associated with a poor prognosis in endometriosis-associated ovarian cancer, Phosphatase and TENsin homolog deleted on chromosome 10 (PTEN)-deficient glioblastomas and renal cell carcinomas (Wakinoue et al., 2018; Chen et al., 2019; Isono et al., 2019). Another ARL4 isoform, ARL4D was first identified as a glioma-associated antigen (Nonaka et al., 2002). Later, a study revealed that ARL4D expression in gliomas is dependent on the loss of PTEN tumor suppressor and consequent activation of the Akt/mammalian Target of Rapamycin (mTOR) pathway (Chi et al., 2008).

### ARL5, ARL6, ARL8, ARL14, and ARFRP1

ARL5A was found to be highly expressed in colorectal cancer and a target of miR-202-3P (Wang et al., 2014). Furthermore, the same study demonstrated that the downregulation of ARL5A and miR-202-3P expression leads to a similar reduction in colorectal cancer cell proliferation (Wang et al., 2014).

In rhabdomyosarcoma, ARL6 was demonstrated to be upregulated in cilia-dependent cancer cells and its silencing decreases cell proliferation (Liu et al., 2016). Additionally, ARL6 downregulation leads to an increase in apoptosis of rhabdomyosarcoma cancer cells due to defects in ciliogenesis and a reduction of Hedgehog (Hh) signaling (Liu et al., 2016).

In the case of ARL8, depletion of ARL8B leads to a reduction in invasion and protease secretion by prostate cancer cells (Dykes et al., 2016). Moreover, ARL8B silencing prevents the growth of prostate tumors in mice (Dykes et al., 2016). Furthermore, the same study revealed that the role of ARL8B in cancer progression is dependent on its function in regulation of lysosomal motility and fusion.

A recent study reported that *ARL14* silencing in lung cancer cells blocks ERK1/2 and p28 signaling and upregulates the cell death inducing DFFA-like effector C (CIDEC), leading to cell cycle arrest (Guo et al., 2019). Also, hypermethylation of *ARL14* was found to be correlated with a poor prognosis of bladder cancer patients (Wang L. et al., 2019).

Finally, in the case of ARFRP1, it was found upregulated in gastric cancer (Mao et al., 2018).

### ARL13B

The role of ARL13B in medulloblastoma and gastric cancer progression, dependent on cilia and Hh signaling was described recently (Bay et al., 2018; Shao et al., 2018). Shao and co-authors showed that ARL13B promotes proliferation, migration and invasion of gastric cancer cells both *in vitro* and *in vivo*, through activation of Smoothened (Smo) and consequent activation of Hh signaling (Shao et al., 2018). In medulloblastoma, ARL13B depletion was reported to lead to a decrease in cilia-dependent oncogenic Hh signaling (Bay et al., 2018). Recently, our group found evidence that ARL13B plays a role in breast tumorigenesis and cancer progression, likely independently of cilia. Indeed, depletion of ARL13B in breast cancer cells leads to a reduction in cell migration and invasion *in vitro* and impaired tumor progression *in vivo* (Casalou et al., 2019). Moreover, our results revealed a new mechanism to explain the role of ARL13B in tumor progression, through the modulation of cell-ECM adhesion and integrin-mediated signaling.

Non-Muscle myosin heavy chain II A (NMIIA) was identified by us as an effector of ARL13B, since it binds to the active form of this protein (Casalou et al., 2014). In the same study, we found that NMIIA mediates ARL13B binding to actin and that both proteins are required for the formation of circular dorsal ruffles (CDRs), which are actin-rich structures required for cell migration (Casalou et al., 2014). Our group also found that GTP-bound ARL13B interacts with NMIIA in breast cancer cells (Casalou et al., 2019). Other studies reported the role of NMIIA in different types of cancers and indicate that *NMIIA* can function as a tumor suppressor or oncogene. For example, NMIIA was found to be overexpressed in gastric cancer (Liu et al., 2012) and promote tumor progression in different types of cancers (Derycke et al., 2011; Katono et al., 2015; Liao et al., 2017; Ye et al., 2017). On the other hand, *NMIIA* was described as a potential tumor suppressor gene in squamous cell carcinomas, since the downregulation of NMIIA associates with poor survival, increased cancer cell invasion and decreased p53 stabilization, *in vitro* and *in vivo* (Schramek et al., 2014). These studies are described in greater detail in two recent reviews (Pecci et al., 2019; Wang et al., 2019a). Other evidence suggests that NMIIA expression is increased in colorectal cancer and that NMIIA enhances tumor aggressiveness through activation of mitogen-activated protein kinase (MAPK) Akt signaling, which promotes EMT (Wang B. et al., 2019). NMIIA was also found to be a promoter of EMT in pancreatic cancer (Zhou et al., 2019). Moreover, in the same study it was observed that NMIIA downregulation results in decreased invasion and metastasis formation through the suppression of canonical Wnt/β-catenin signaling (Zhou et al., 2019).

### TRIM23 and SAR1

In hepatocellular carcinomas, miR-194, which targets TRIM23 was found to be downregulated. Moreover, overexpression of miR-194 decreases cell migration, invasion and metastasis of hepatocellular carcinoma cells, through inhibition of Nuclear Factor (NF)-κb activity (Bao et al., 2015). Furthermore, TRIM23 was found to be overexpressed in gastric cancer, both in cell lines and tissues (Yao et al., 2018).

In the case of SAR1, it was found to be overexpressed in liver cancer (He et al., 2002). More recently, SAR1b was identified as a promoter of drug resistance, namely mTOR Complex (mTORC) inhibitors, in liver tumor initiating stem cells and hepatocellular carcinoma cells (Wu R. et al., 2018).

## Arf Family Proteins and Their Activity Regulators and Effectors That Can Function as Tumor Suppressor Genes

In some cases, the expression of membrane traffic regulators, namely ARF family proteins or their effectors, GEFs or GAPs is found downregulated in tumor cells (Tables 1, 2).

### ARF3

Besides its behavior as an oncogene in breast cancer, ARF3 has been found downregulated in gastric cancer (Chang et al., 2009). In fact, ARF3 expression is significantly decreased in gastric cancer stages I-III, when compared with paired normal gastric mucosa tissues, indicating that this protein could be a marker for gastric cancers without metastasis. The clinical significance of these results remains to be elucidated.

### ARF GEFs and GAPs

SMAP1 or ARFGAP1 is a member of the ARF GAP family that is involved in clathrin-dependent endocytosis of the Transferrin receptor and E-cadherin (Kon et al., 2008; Kobayashi et al., 2014). In colorectal cancers with microsatellite instability, short deletions or insertions frequently occur in *SMAP1*, generating a premature termination codon. This results in reduced or abolished SMAP1 protein levels in colorectal tumors (Kon et al., 2014).

Unlike other phosphotyrosine proteins that are usually overexpressed or hyperphosphorylated in gastric tumor cells, the ARF GAP ARAP3 is downregulated in gastric cancer tissues (Yagi et al., 2011). Furthermore, GIT2 stabilizes FAs by reducing Rac1 activity in the breast cancer cell line MDA-MB-231 (Frank et al., 2017). Also, in a gene expression profile analysis of breast cancer patient samples, GIT2 was found downregulated in a group of lymph node-positive breast cancer patients (Sirirattanakul et al., 2015).

Finally, EFA6 GEFs are downregulated in breast, brain and ovarian cancers (Pils et al., 2005; Van Den Boom et al., 2006; Zangari et al., 2014).

### ARL2

ARL2 has been shown to directly influence α/β-tubulin polymerization in the breast cancer cell line MCF-7 (Beghin et al., 2007). Moreover, MCF-7 cells expressing higher levels of ARL2 are more sensitive to cytotoxic agents, while cells with reduced expression of ARL2 show enhanced resistance to the same agents (Beghin et al., 2008). This resistance is mediated by Protein Phosphatase 2A (PP2A), whose activity is regulated by ARL2. When ARL2 is decreased, impaired dephosphorylation of p53 by PP2A occurs, leading to an increase of phosphorylated p53, which alters PP2A localization and causes a chemo-resistant phenotype (Beghin et al., 2008). Moreover, *in vitro* assays using breast cancer cells depleted for ARL2 show less contact inhibition, an enhanced clonogenic potential and increased proliferation than control cells (Beghin et al., 2009). Furthermore, using orthotopic mouse models, depletion of ARL2 was shown to impair cancer progression (Beghin et al., 2009). Additionally, ARL2 downregulation was recently correlated with more aggressive cases of glioma and a lower survival of the patients (Wang et al., 2018). Finally, ARL2 overexpression inhibits proliferation, as well as migration and tumorigenicity of glioma cells, through regulation of the receptor tyrosine kinase AXL, a known regulator of glioma tumorigenesis (Wang et al., 2018).

### ARL3, ARL4, and SAR1

*ARL3* mRNA and protein expression were shown to be downregulated in gliomas (Wang et al., 2019b). Furthermore, an extensive bioinformatics analysis suggested that ARL3 plays a role in angiogenesis and immune cell infiltration in the tumor microenvironment (Wang et al., 2019b).

ARL4C was associated with reduced metastatic potential of ovarian cancer cells, in which it inhibits cell motility but not cell proliferation (Su et al., 2015). Furthermore, *ARL4C* mRNA expression is lower in ovarian cancer samples of patients with a poor treatment response, while patients with higher ARL4C expression show increased overall survival (Su et al., 2015).

Finally, SAR1B was identified as a potential metastatic suppressor in colorectal cancer, through a targeted proteomic approach (Huang and Wang, 2019). Also, migration and invasion assays showed that SAR1B silencing leads to an increase in colorectal cancer cell motility and invasive capacity (Huang and Wang, 2019).

### ARL11

*ARL11*, also known as ADP Ribosylation factor-Like Tumor Suppressor gene 1 (*ARLTS1*) was described as a potential low-penetrance tumor suppressor gene in different types of cancers, such as breast cancer, melanoma and chronic lymphocytic leukemia (Calin et al., 2005). Different variants of *ARLTS1* have been associated with familial and sporadic cancers, where the mutations Trp149Stop and Cys148Arg are the most studied (Yendamuri et al., 2008). The nonsense mutation Trp149Stop leads to the production of a truncated protein unable to bind GTP, which results in decreased apoptotic potential of the cell (Petrocca et al., 2006). Both variants were found to be associated with predisposition to familial breast cancer and, more recently to familial hematological malignancies (Calin et al., 2005; Frank et al., 2006b; Hamadou et al., 2017). Additionally, the Cys148Arg variant was associated with melanoma and both familial and sporadic colorectal cancers (Frank et al., 2006a, c; Castellví-Bel et al., 2007). Furthermore, *ARLTS1* expression was found to be decreased in different types of tumors, including ovarian, lung and prostate cancer, as well as chronic lymphocytic leukemia (Yendamuri et al., 2008; Siltanen et al., 2013). More recently, a study in ovarian cancer suggested that ARLST1 increases tumor cell sensitivity to chemotherapeutic agents by regulating apoptosis (Yang et al., 2011).

## Therapeutic Strategies

**FIGURE 1:**
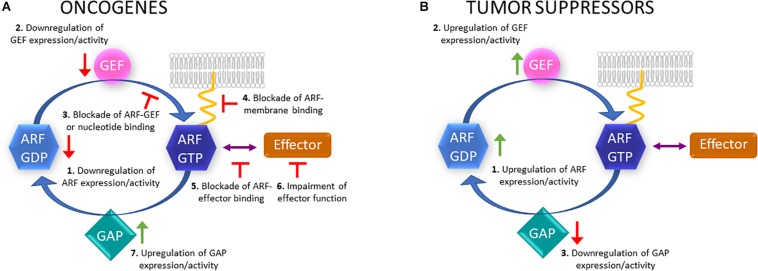
Putative therapeutic strategies to target ARF proteins, GEFs, GAPs and effectors. **(A)** In the case of ARF family members that can act as oncogenes, their expression or activity could be downregulated (1); GEF activity or expression downregulated (2); ARF-GEF binding blocked or nucleotide binding blocked (3); active ARF binding to membranes (4) or effectors blocked (5); effector function impaired (6); GAP expression or activity upregulated (7). **(B)** Regarding ARF proteins that can act as tumor suppressors, their expression or activity could be upregulated (1); GEF activity or expression upregulated (2); GAP activity or expression downregulated (3).

As can be concluded from Tables 1, 2, several ARFs and ARF GEFs and GAPs are overexpressed in different types of cancers. Therefore, therapeutic strategies aiming to inhibit the expression of these proteins can be proposed. Other approaches like the use of small GTPase inhibitors that impair GTP binding or the binding to membranes, the blockade of GEF activity or ARF-GEF interaction, should also be considered. Furthermore, the stimulation of GAP activity/expression and the inhibition of the interaction with downstream effectors or the function/expression of these effectors can also be envisaged (Figure 1). For instance, the inhibitor LM11 can abolish specifically ARF1 activation through the blockade of the binding of the ARF GEF Cytohesin 2/ARNO (Flisiak et al., 2008; Xie et al., 2016). Indeed, it has been shown that the aggressiveness of breast tumors that overexpress ARF1 is reduced after treatment with this inhibitor through the decrease in cell invasion and proliferation and increased apoptosis (Schlienger et al., 2015; Xie et al., 2016). Also, the small inhibitor EXO2 reduces ARF1 activation and effectively impairs the proliferation of prostate cancer cells by blocking ERK1/2 activation (Lang et al., 2017). Moreover, EXO2 inhibits invasion of prostate cancer cells and induces their apoptosis. Furthermore, the same study shows that the simultaneous blockade of ARF1 and RAS activation in prostate cancer is a potential targeted strategy to prevent the development of this type of tumor (Lang et al., 2017).

Since some ARF proteins like ARF6 and ARF1, are ubiquitously expressed and perform essential functions in all cell types (D’Souza-Schorey and Chavrier, 2006), targeting the proteins themselves could have dramatic and unwanted consequences. In alternative, targeting their regulators, such as ARF GEFs or GAPs, might represent a viable strategy for the development of specific anti-cancer therapies. Regarding the targeting of ARF GEFs, it has been shown that SecinH3, an ARF GEF inhibitor that impairs both ARF1 and ARF6-dependent signaling, is effective in decreasing the growth of breast cancer xenografts and reducing lung metastasis (Zhao et al., 2016), while suppressing angiogenesis of melanoma and lung carcinoma tumors (Grossmann et al., 2014; Hongu et al., 2015). Thus, inhibitors of the ARF6-dependent signaling pathway could be useful to control specifically tumor invasion and angiogenesis.

It has been observed that several ARF GAPs are overexpressed in cancer (Table 2), even though overexpression of ARF GAPs does not imply increased GAP activity. For instance, AGAP2 expression in chronic myeloid leukemia cells and prostate cancer is regulated by Specific Protein 1 (SP1) and ATRA (Doush et al., 2019). Additionally, the authors observed that the treatment of cells of these types of cancer with the polyphenol curcumin, leads to a decrease in ATRA-mediated AGAP2 expression (Doush et al., 2019; Giordano and Tommonaro, 2019). This data illustrates the relevance of regulating ARF GAP expression levels in cancer. On the other hand, it was observed that QS11, the only inhibitor of ARF GAPs known, binds to ARF GAP1 and inhibits the activity of this GAP on ARF1 and ARF6 (Zhang et al., 2007; Zhu et al., 2012). Interestingly, it was observed that QS11 blocks migration of metastatic breast cancer cells, *in vitro* (Zhang et al., 2007). Thus, inhibitors of ARF GAP activity could also be effective in controlling cancer cell migration and invasion.

Inhibition of the expression or function of downstream effectors of ARF family proteins is also a plausible strategy to impair the oncogenic potential of ARFs and ARLs. An interesting candidate is NMII. Indeed, several types of cancer exhibit differential expression and/or activation of NMII isoforms, leading to alterations in cell migration and invasion that are involved in tumorigenesis (Newell-Litwa et al., 2015). Moreover, we found that NMIIA is an effector of ARL13B (Casalou et al., 2014, 2019). Furthermore, blebbistatin inhibits the ATPase activity of NMIIA and has been found to block invasiveness of both breast cancer cells (Derycke et al., 2011) and pancreatic adenocarcinoma cells (Duxbury et al., 2004), and is phototoxic in human cancer cells under exposure to blue light (Mikulich et al., 2012).

## Conclusion and Perspectives

Members of the RAS superfamily of small GTPases are master regulators of all the steps involved in membrane traffic. Thus, it is not surprising that many of them are hijacked by cancer cells to enhance their capacity to form a tumor and spread to other organs. In particular, ARF family proteins, their GEFs, GAPs and effectors are often upregulated in expression and/or activity in several types of cancer. Moreover, upregulated expression/activity can be linked to enhanced cancer progression and aggressiveness. Therefore, these proteins are good candidates to serve as therapeutic targets and, indeed several strategies have already been proposed and tested. These include the targeting of ARF proteins themselves or their GEFs, GAPs or effectors. While our knowledge of the GEFs, GAPs and effectors of ARFs is fairly complete, much less is known about the functions and identity of GEFs, GAPs and effectors of ARL subfamily members. Hence, the knowledge about these molecular players should be developed in order to find new therapeutic strategies for cancer types where ARLs or their regulators/effectors are subverted. Since most ARF family proteins are ubiquitous and required for essential cellular functions, the targeting of specific effectors and GEFs/GAPs could ensure tissue/function specificity. Nevertheless, specificity could also be achieved through targeted delivery of vectors/drugs.

In conclusion, the study of the mechanisms subverted by cancer cells involving ARF family proteins and their regulators of activity and effectors can shed light on the functions of these proteins and simultaneously provide clues about new therapeutic targets and strategies, which continue to be a pressing need in the cancer field.

## Author Contributions

All authors conceived, wrote, reviewed, and edited the manuscript.

## Conflict of Interest

The authors declare that the research was conducted in the absence of any commercial or financial relationships that could be construed as a potential conflict of interest.

## Abbreviations

4-HPR: N-(4-hydroxyphenil retinamide)
Akt: Protein kinase B
ARF: ADP-Ribosylation Factor
ARL: ARF-Like
ARLTS1: ADP-Ribosylation factor-Like Tumor Suppressor gene 1
ATRA: All-*Trans* Retinoic Acid
BART: Binder of ARL Two
Bax: B-cell lymphoma 2-Associated X protein
CDR: Circular Dorsal Ruffle
CIDEC: Cell Death Inducing DFFA-like Effector C
ECM: ExtraCellular Matrix
EGF: Epidermal Growth Factor
EGFR: Epidermal Growth Factor Receptor
EMT: Epithelial-Mesenchymal Transition
ER: Endoplasmic Reticulum
ERK: Extracellular signal-Regulated Kinase
FA: Focal Adhesion
FAK: Focal Adhesion Kinase
FOXO1: Forkhead bOX O1
GAP: GTPase-Activating Protein
GDP: Guanosine DiPhosphate
GEF: Guanine nucleotide Exchange Factor
GTP: Guanosine TriPhosphate
HER2: Human Epidermal growth factor Receptor 2
HGF: Human Growth Factor
Hh: Hedgehog
IGFR: Insulin Growth Factor Receptor
MAPK: Mitogen-Activated Protein Kinase
miRs: microRNAs
mTOR: mammalian Target of Rapamycin
mTORC: mammalian Target of Rapamycin Complex
NF: Nuclear Factor
NMIIA: Non-Muscle Myosin heavy chain II A
PAK: p21-Activated Kinase
PI: PhosphoInositide
PI3K: PhosphoInositide 3-Kinase
PI3KCD: PhosphoInositide 3-Kinase Catalytic subunit Delta
PIX: PAK- Interacting eXchange factor
PLD: PhosphoLipase D
PMA: Phorbol-12-Myristate 13-Acetate
PP2A: Protein Phosphatase 2A
PTEN: Phosphatase and TENsin homolog deleted on chromosome 10
Rho: RAS homolog gene family member
Rac: RAS-related C3 botulinum toxin substrate
ROS: Reactive Oxygen Species
SAR: Secretion-Associated RAS-related
Smo: Smoothened
SP1: Specificity Protein 1
Src: Rous Sarcoma oncogene cellular homolog
TET: Ten-Eleven Translocation methylcytosine dioxygenase
TRIM23: TRIpartite Motif-containing protein 23
UCA1: Urothelial Cancer Associated 1.
